# Acral reed nevus with parallel ridge pattern: an exception to the rule of malignancy^☆^

**DOI:** 10.1016/j.abd.2021.05.022

**Published:** 2023-01-03

**Authors:** Elena Canal-Garcia, Xavier Soria, Felip Vilardell, Rosa M. Marti

**Affiliations:** a Department of Dermatology, Hospital Universitari Arnau de Vilanova, University of Lleida, IRBLleida, Lleida, Spain; b Department of Pathology, Hospital Universitari Arnau de Vilanova, University of Lleida, IRBLleida, Lleida, Spain; c Centre of Biomedical Research on Cancer, Instituto de Salud Carlos III, Madrid, Spain

Dear Editor,

Reed nevi (RNs) are almost exclusively junctional neoplasms distinguished as a variant of Spitz nevus (SN) by their significant melanogenesis and growth pattern. Acral presentation of SN is rare and has specific clinical and histopathological features.^1^ Nonetheless, dermoscopic findings of SN and its variants on the acral skin are poorly documented.^2^ Herein we describe a case of RN on the volar skin of a finger in which parallel ridge pattern was observed by dermoscopic examination.

A 9-year-old boy presented with an asymptomatic pigmented lesion on his right hand. He had detected the lesion 8 months previously and had enlarged gradually. He had no personal or family history of malignant tumors. Physical examination revealed an asymmetrical dark brown macule, 13×3 mm in size, located on the volar region of the second right finger. The lesion exhibited an atypical linear morphology similar to a crescent moon (Fig. 1A). Dermoscopic findings showed a brownish parallel ridge pattern with some streaks at the periphery (Fig. 1B). A complete surgical excision was done to rule out malignancy. Histological examination revealed several small nests, vertically oriented, composed of heavily pigmented spindled melanocytes along the dermo-epidermal junction (Fig. 1C‒D). No melanocytes were seen in the dermis. A diagnosis by an RN was done.

**Figure 1:**
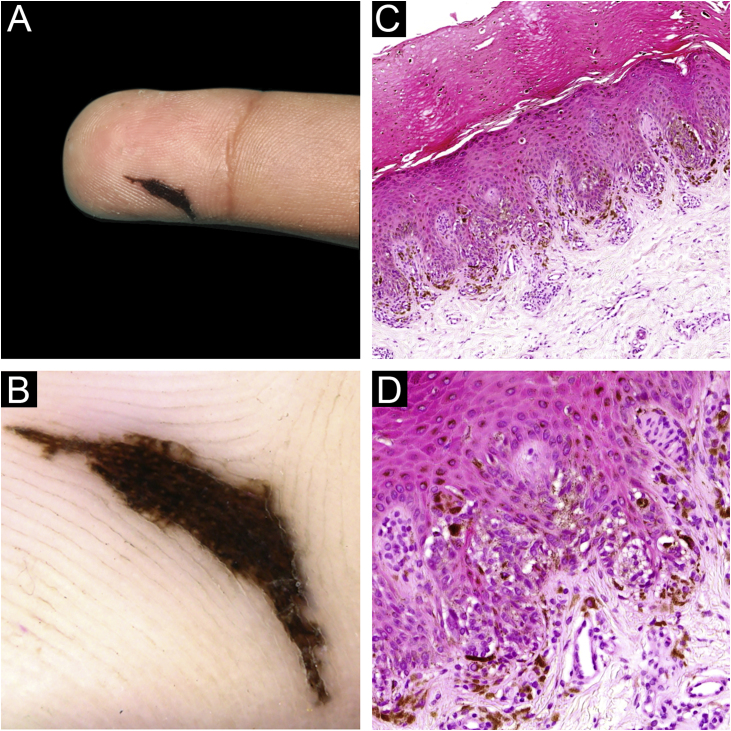
Reed nevus on the finger (A) Clinical features of the lesion. (B) Dermoscopic findings show a parallel ridge pattern with some peripheral streaks. (C) Histopathological findings were tumor cell nests scattered within the epidermis (Hematoxylin & eosin, ×100). (D) Junctional nests were composed of heavily pigmented spindle-shaped melanocytes, vertically oriented (Hematoxylin & eosin, ×200).

Acral SNs understood by those located on soles, palms and fingers are infrequent, being reported to comprise less than 2% of all SNs.^1^ They are more common in young female adults, more frequently located on the feet, and larger than acral melanocytic nevi.^1^ Acral RN or “pigmented spindle-cell nevus” is the most common variant of SN that typically presents as a heavily pigmented lesion.^1^ Given the overlapping clinicopathologic features with malignancy, their clinical diagnosis is often of an atypical nevus or a malignant melanoma.^1^, ^2^ Dermoscopic examination is useful in differentiating RN and acral lentiginous malignant melanoma.^2^ Dermoscopic patterns most commonly associated with SN are starburst and globular patterns.^2^ However, a characteristic pattern upon dermoscopic examination of SN localized on the glabrous skin has not been described.^2^, ^3^ A review of the literature revealed only nine cases of acral SN and its variants with the dermoscopic examination.^4^ A parallel furrow pattern with peripheral dots, streaks, and projections and a starburst pattern has been reported in four and three cases, respectively.^4^ A crista-dotted pattern was described in one case of SN on the palm.^3^ Only Jurakić et al. in 2018 reported a young female with a rapidly growing plantar pigmented SN that had a dermoscopic parallel ridge pattern with few peripheral globules, similar to our case.^5^

In conclusion, we report a case of an acral RN showing a parallel ridge pattern on dermoscopy, an exception to the rule of malignancy. The present report aims to highlight that, although such a pattern is highly suggestive of melanoma, it can also be seen in a proportion of acral SNs localized on the glabrous skin. However, an SN with asymmetry and/or atypical dermoscopic pattern is impossible to differentiate from melanoma and, thereby, must be excised irrespective of age or clinical morphology.

## Financial support

None declared.

## Authors’ contributions

Elena Canal-Garcia: Study concept and design; drafting and editing of the manuscript; writing of the manuscript or critical review of important intellectual content; data collection, analysis and interpretation; effective participation in the research guidance; intellectual participation in the propaedeutic and/or therapeutic conduct of the studied cases; critical review of the literature; final approval of the final version of the manuscript.

Xavier Soria: Study concept and design; drafting and editing of the manuscript; writing of the manuscript or critical review of important intellectual content; data collection, analysis and interpretation; effective participation in the research guidance; intellectual participation in the propaedeutic and/or therapeutic conduct of the studied cases; critical review of the literature; final approval of the final version of the manuscript.

Felip Vilardell: Writing of the manuscript or critical review of important intellectual content; data collection, analysis and interpretation; effective participation in the research guidance; intellectual participation in the propaedeutic and/or therapeutic conduct of the studied cases; critical review of the literature; final approval of the final version of the manuscript.

Rosa M. Martí: Study concept and design; drafting and editing of the manuscript; writing of the manuscript or critical review of important intellectual content; data collection, analysis and interpretation; effective participation in the research guidance; intellectual participation in the propaedeutic and/or therapeutic conduct of the studied cases; critical review of the literature; final approval of the final version of the manuscript.

## Conflicts of interest

None declared.
